# Situation actuelle de la schistosomiase dans l'aire de santé de Santchou, (District de santé de Santchou, Région de l'Ouest-Cameroun)

**DOI:** 10.11604/pamj.2016.24.137.8778

**Published:** 2016-06-10

**Authors:** Huguette Nguedie Tchouanguem, Florent Ymele Fouelifack, Basile Keugoung, Loic Dongmo Fouelifa, Roger Somo Moyou

**Affiliations:** 1 Hôpital de District de Biyem Assi, Yaoundé-Cameroun; 2 Hôpital Central de Yaoundé, Cameroun; 3 Groupe Associatif pour la Recherche, l'Education et la Santé (GARES-Falaise), Dschang, Cameroun, Ministère de la Santé Publique, Yaoundé, Cameroun; 4 Faculté des Sciences de la Santé, Université de Lomé, Ecole des Services de santé des Armées de Lomé, Togo; 5 Département de Santé publique, Faculté de Médicine et des Sciences Biomédicales de l'Université de Yaoundé I, Ministère de la Recherche Scientifique et de l'Innovation, Yaoundé, Cameroun

**Keywords:** Situation actuelle, schistosomiase, aire de santé de Santchou, Cameroun, current situation, schistosomiasis, Santchou health area, Cameroon

## Abstract

**Introduction:**

La schistosomiase, deuxième endémie parasitaire mondiale, est une parasitose due aux trématodes du genre Schistosoma. Nos objectifs étaient d’évaluer les prévalences des différentes espèces de schistosomes (Schistosoma mansoni, haematobium, et intercalatum) chez les écoliers, et d'identifier les facteurs de risques, les signes cliniques de la schistosomiase, et les mollusques hôtes intermédiaires des schistosomiases dans les eaux stagnantes.

**Méthodes:**

L’étude était transversale et s'est déroulée sur 3 mois. Elle consistait en l'enregistrement des données sociodémographiques et cliniques, le prélèvement des échantillons de selles et d'urines, la recherche des mollusques et le traitement des écoliers positifs à d'autres helminthes. Les examens de laboratoire se sont déroulés à l'Institut de recherches Médicales et d’études des Plantes Médicinales à Yaoundé où on examinait les échantillons de selles et d'urine par les méthodes de KATO KATZ et de centrifugation respectivement, et un malacologiste déterminait l'espèce des mollusques.

**Résultats:**

Au total, 400 élèves âgés entre 8-16 ans, soit 223 (55.7%) filles et 177 (44.3%) garçons répartis dans 4 écoles primaires ont participé à l’étude. L'enquête sociale a révélé que 154 écoliers sur 400 (soit 38.5%) étaient en contact avec les eaux de rivière au moins une fois par semaine, dont 58% aux environs de midi. Tous les élèves avaient au moins un signe de schistosomiase bien que non spécifique prédominé par des douleurs abdominales à 72% (n= 288 sur 400). Sur le plan biologique aucun œuf de schistosomiase n'a été mis en évidence. Le taux d’émission de cercaire était négatif chez les 100 espèces aquatiques retrouvées.

**Conclusion:**

L'aire de santé de Santchou n'est pas un foyer actif de schistosomiase, mais reste une zone à risque du fait de la riziculture et de la présence des eaux stagnantes. L'intensification des campagnes d’éducation sanitaire dans la population permettrait de retarder la survenue de cette parasitose dans la localité.

## Abstract (en)

**Introduction:**

Schistosomiasis, the second endemic parasitic infection in the world, is a parasitosis caused by trematodes from the genus Schistosoma. Our study aims to assess the prevalence of different species of schistosomes (Schistosoma mansoni, haematobium and intercalatum) among schoolchildren and to identify risk factors, clinical signs of schistosomiasis, and schistosomiasis intermediate host snails in stagnant water.

**Methods:**

We conducted a cross sectional study over a three months period. The study consisted of sociodemographic and clinical data recording, collection of stool samples and urine, molluscan research and treatment of positive students for other helminths. Laboratory tests were performed at the Medical Research Institute and the study of Medicinal Plants in Yaounde where stool samples and urine were examined using KATO KATZ and centrifugation technique respectively, and shellfish species were determined by a malacologist.

**Results:**

A total of 400 students aged between 8-16 years, 223 (55.7%) girls and 177 (44.3%) boys attending 4 elementary school were enrolled in the study. The social survey revealed that 154 students out of 400 (or 38.5%) were in contact with the river water at least once a week, 58% from around noon. All students had at least one symptom of schistosomiasis although nonspecific and dominated by abdominal pain in 72% of cases (n = 288 of 400). Biologically, no schistosomiasis eggs were detected. Cercaria releasing rate was negative in the 100 watery species found.

**Conclusion:**

The Santchou health area is not an active outbreak of schistosomiasis, but remains a risk area because of rice cultivation and stagnant water. The intensification of health education campaigns among the general population would delay the onset of this infection in the locality.

## Introduction

La schistosomiase (encore appelée schistosomose ou bilharziose) est une maladie parasitaire due aux trématodes du genre Schistosoma (S.), donc 5 espèces sont pathogènes pour l'homme. C'est la deuxième grande endémie parasitaire dans le monde. On estime à environ 4 millions d'infestations par an et entre 300000 à 500000 décès par an. Le facteur influençant le développement de la schistosomiase est le contact avec les gites des mollusques et larves (eaux douces infectées). Des 5 espèces pathogènes pour l'homme, 3 sont présentes au Cameroun: S. haematobium agent de la schistosomiase uro-génitale, S. mansoni agent de la schistosomiase intestinale et hépato splénique, et S. intercalatum agent de la schistosomiase rectale. Au Cameroun, l'incidence est de 1000 à 1500 cas par an. En l'an 2000 on notait environ 1,7 million de personnes infectées [1] de schistosomiase dont 80% des cas dans les régions septentrionales [2]. Santchou, localité autrefois aménagée de grandes surfaces de riziculture irriguée est une zone potentielle de développement de la schistosomiase démontrée par une étude en 2002 [3]. Notre étude avait pour objectifs d’étudier la situation actuelle de la schistosomiase dans la localité de Santchou, plus spécifiquement d’évaluer les prévalences de S. mansoni, de S. haematobium et/ou de S. intercalatum, d'identifier les facteurs de risques et les signes cliniques de la schistosomiase chez les écoliers, et de rechercher les mollusques hôte intermédiaire des schistosomiases dans les eaux stagnantes de la localité de Santchou.

## Méthodes

L’étude était transversale sur 3 mois (de septembre à novembre 2011) dans 4 écoles primaires proches des cours d'eau, dans l'aire de Santé de Santchou. L'aire de Santé de Santchou fait partie du district de santé de Santchou, dans l'arrondissement de Santchou, département de la Menoua, région de l'Ouest- Cameroun. L'arrondissement de Santchou s’étend sur une superficie de 355 km^2^. Son Relief est fait de plaines. Les inondations sont fréquentes en saisons de pluies. Les cours d'eau débordent les ruelles, souillent les eaux des rivières à usage domestique et exposent les populations à des maladies. Sa population est d'environ 26085 habitants. La principale activité de la population est l'agriculture et l’élevage. La riziculture ayant débuté en 1973 a été arrêtée en 1987 pour des raisons économiques [4, 5]. Notre population d’étude était des élèves des 4 écoles primaires proches des cours d'eaux de la ville de Santchou (Cours moyens 1 et 2). Etait exclus de l’étude, tout écolier ayant pris un antihelminthique dans les 3 derniers mois, ou n'ayant pas donné son consentement. Le choix de la population a été fait sur la base que la tranche d’âge la plus touchée selon une étude antérieure variant entre 10-14 ans [6].

### Procédure

Après avoir sélectionné les participants remplissant les critères d'inclusion à l’étude, les techniques de prélèvement leur étaient expliquées. Deux pots leurs étaient remis, l'un pour les selles et l'autre pour les urines. Une enquête sociale puis clinique était faite pour rechercher les facteurs de risque, des signes et symptômes en rapport avec la schistosomiase. Toutes ces variables étaient mentionnées sur une fiche technique préétablie. Les échantillons de selles étaient conservés dans des glacières pendant toute la durée des prélèvements. L'urine était conservée avec 2-3 millilitres de formol dilué à 10%. La collecte systématique des mollusques se déroulait dans les différents points d'eau, à l'aide d'une épuisette à longue manche car les gites étaient parfois inondés d'eau de pluies. Les mollusques étaient par la suite, prélevés avec une pince et mis dans des boîtes à pétri contenant du coton imbibé d'eau de leur gite. Ils étaient par la suite transportés au laboratoire de parasitologie de l'Institut de Recherche Médicales et d’études des Plantes Médicinales de Yaoundé pour déterminer pour déterminer l'espèce du mollusque et faire le test d’émission de cercaire, tout ceci dans le but de rechercher les formes infestantes de schistosomes. Les selles étaient examinées au laboratoire, par la méthode d'enrichissement (méthode de KATO-Katz) [7] et les urines par la méthode de centrifugation. Les lames étaient lues au microscope à l'objectif 10 puis à l'objectif 40. La technique de KATO-KATZ est une méthode semi-quantitative plus adaptée à l’étude de la schistosomiase intestinale [8]. Pour déterminer la taille minimale de notre échantillon, nous avons utilisé la formule de LORENTZ: N = p (1 – p) (Zα/d^2^) P = prévalence de la dernière étude dans la localité ou une localité très proche en 1997: 26% à Nkonsoung [9] Zα = erreur d’échantillonnage elle est de 1.96 pour a =5% (0.05) d = degré de confiance (0.05) N= 0.26 (1 -0.26) (1.96/0.05)2 = 296 sujets. Pour augmenter la puissance de notre étude, nous avons recruté 400 sujets. Pour les mollusques, nous avons ramassés 100 mollusques dans les cours d'eaux à courant lent.

### Analyse des données

Les données étaient saisies sur le logiciel Excel version Windows 2007 et analysées grâce au logiciel Epi Info 3.5.1. Le test de Student était utilisé pour la comparaison des moyennes et le khi-deux pour l'indépendance et les liaisons entre les données statistiques. La liaison était considérée significative pour toute valeur p inférieure à 0.05.

### Considérations éthiques

La clairance éthique N° 147/CNE/SE/2011 avait été au préalable obtenue auprès du comité national d’éthique du Cameroun. Un formulaire de consentement éclairé était lu et signé par les parents ou tuteurs des écoliers. Des autorisations étaient obtenues auprès des autorités administratives, sanitaires et académiques de l'arrondissement de Santchou. Les informations recueillies ont été traitées de façon confidentielle. Après les résultats, une seconde descente a été faite pour rendre compte aux autorités et traiter gratuitement les écoliers positifs à d'autres helminthiases à base d'albendazole.

## Résultats

Au total 400 élèves avaient été examinés. Cet effectif provenait de: l’école catholique de Santchou (96 élèves), l’école publique bilingue de Santchou (94 élèves), l’école publique de Santchou groupe I (107 élèves) et l’école publique de Santchou groupe II (103 élèves).

### Profil sociodémographique

#### Répartition de la population par sexe et par tranche d’âge

La répartition des élèves par sexe et par groupe d’âge est représentée dans le Tableau 1.

**Tableau 1:** Répartition de la population par sexe et par tranche d’âge

	Féminin		Masculin		Total	
	Effectif	%	Effectif	%	Effectif	%
[8 - 12 ans [	169	42,3	110	27,5	279	69,8
[12 - 15 ans [	47	11,7	59	14,8	106	26,5
≥ 15 ans	7	1,8	8	2,0	15	3,8
**Total**	223	55,7	177	44,3	400	100,0

### Enquête sociologique

#### Répartition des participants selon le contact ou non avec les eaux de rivières

Sur 400 écoliers, 154 (soit 38.46%) étaient régulièrement (au moins une fois par semaine) en contact avec les eaux des rivières, ceci pendant la lessive, dans les champs ou au cours des jeux tel que le football.

#### Répartition des participants en contact avec l'eau, selon l'heure de contact

**Figure 1:**
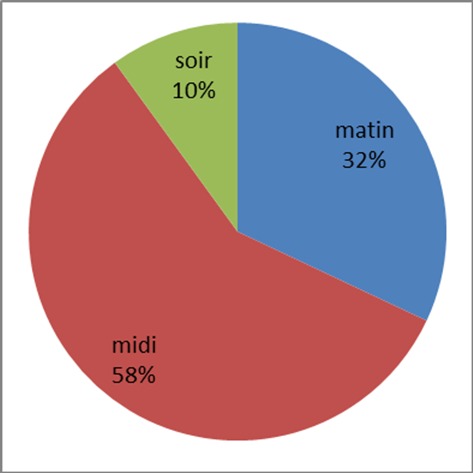
Répartition des participants en fonction du moment du contact avec l'eau

La répartition des participants régulièrement en contact avec l'eau, selon l'heure de contact avec les eaux de rivières est représentée par la Figure 1.

### Enquête clinique

#### Répartition des différents signes et symptômes cliniques de schistosomiase trouvés chez les écoliers

**Figure 2:**
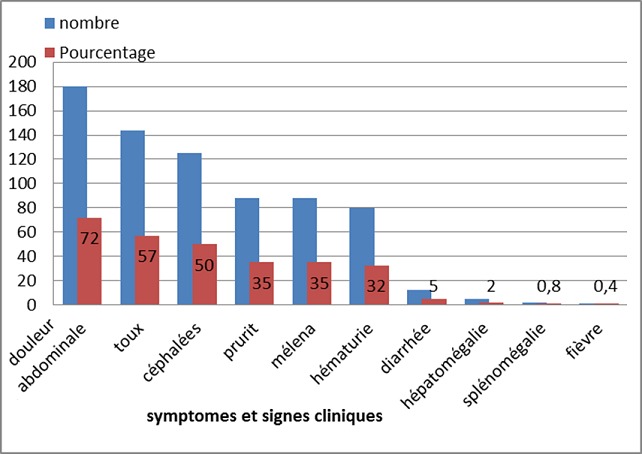
Répartition des différents symptômes et signes cliniques retrouvés chez les participants

Les différents signes et symptômes de schistosomiase trouvés chez les écoliers sont représentés dans la Figure 2.

### Enquête biologique

#### Recherche des œufs de schistosome dans les selles/urines

**Figure 3:**
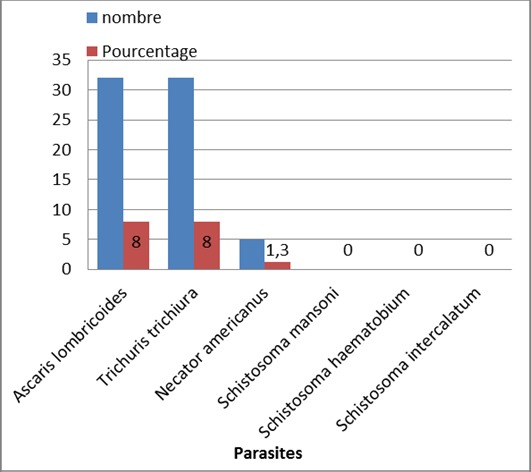
Prévalences des parasites dans les selles/urines

Des 400 participants, la prévalence des parasites dans les selles et/ou urines est représentée dans la Figure 3.

### Enquête malacologique

Malgré les difficultés liées aux fortes pluies qui rendaient difficile l'accès aux éventuels gites, nous avons récoltés 125 mollusques parmi lesquelles 100 étaient des espèces aquatiques. Le test d’émission des cercaires de ces espèces aquatiques était négatif.

## Discussion

Nous avons été limités dans la récolte des mollusques par des fortes pluies qui rendaient inaccessibles certains gites éventuels. Des études antérieures ont montré que les zones de riziculture irriguées étaient favorables à l'installation et à l'extension des schistosomiases [3, 10–13]. La riziculture en elle-même n'est pas un environnement favorable pour développer des mollusques, mais la transmission dans la région est le plus souvent secondaire à des canaux d′irrigation. Les fossés obstrués par les plantes sont les endroits où les mollusques se développent le mieux. Bulinus spp. est retrouvé en grand nombre dans les parties basses du réseau aquatique. Ils survivent aux conditions de sécheresse en s'enfuyant dans la boue. Les systèmes d′irrigation des constructions récentes ne sont pas massivement envahis par les mollusques. La prévalence de la schistosomiase dans une zone correspond à la distribution des hôtes intermédiaires des schistosomes et de la densité des mollusques [12]. L’étude faite à Yagoua I sur 990 échantillons (provenant de 4 villages donc 3 dans la zone de riziculture et 1 hors de cette zone) montre que la prévalence de la schistosomiase et la charge parasitaire varie d'un village à l'autre, en relation avec la distance entre le village et le réseau d'eau d'irrigation de la riziculture. Les riziculteurs sont plus exposés que les non-riziculteurs [13]. Mais nous n'avons pas retrouvé des cas d'infestation à schistosome dans notre échantillon. Les études faites dans les zones irriguées ont donné de fortes prévalences de schistosomiases [14], motivant la localité de Santchou comme lieu de notre étude. Cette localité est une zone ancienne de riziculture mais d'irrigation permanente qui regorgent une grande population, avec des comportements à risques: 154 élèves sur 400 (soit 38.46%) se baignent dans les rivières, des signes cliniques évocateurs et l’écologie propice. L'exposition de ces écoliers se fait majoritairement à midi (Figure 1) concordant bien avec l'heure propice à l’émission des cercaires.

### Les symptômes et signes cliniques

Sur le plan clinique les renseignements regroupés corroborent avec ceux des études précédentes malgré les résultats négatifs pour les schistosomes (Figure 2). Ces signes: douleurs abdominales à 72%, toux à 57%, céphalées à 50%, sont en rapport avec d'autres parasitoses telles que les helminthiases donc la prévalence est de 17.33% et le paludisme. Contrairement à l'hématurie qui est faiblement représentée dans d'autres études [15], nous avons eu une prévalence de 32%. Nous n'avons eu aucun cas d'anémie, d'ascite, d'hypertension portale ou de circulation collatérale qui sont généralement observés dans les cas d'hyperparasitémie bilharzienne. Mais nous avons eu 5 cas de d'hépatomégalie et 2 cas de splénomégalie qui est un signe en étroite relation avec la schistosomiase intestinale seulement chez les adultes [16].

### Enquête biologique

Le choix de l'examen a été basé sur l’étude faite à Yagoua I montrant que la prévalence donnée par le test d'hémmaglutination est inférieure à celle donnée par les examens d'urine. Il est préférable de considérer la prévalence donnée par les examens d'urine. Sur le plan clinique, le nombre d’œufs est en rapport avec la gravité de la maladie. Sur le plan épidémiologique ce sont les sujets émettant les œufs, qui transmettent la maladie [13]. L'absence d’œufs dans les selles des écoliers de Santchou serait due au fait que le déparasitage est devenu systématique dans les milieux scolaires diminuant ainsi la charge parasitaire. Les cours d'hygiène sont de règles dans toutes les écoles. Tout ceci nous conduit à des résultats négatifs car il existe un seuil de détermination d’œuf dans les selles. Notre étude a montré un pourcentage de 8% pour les 2 parasitoses (Figure 3). Néanmoins nous notons aussi la présence de *Necator americanus* à 1.33%. Une étude a retrouvé ces géo helminthes [9].

## Conclusion

La prévalence de la schistosomiase à Santchou était nulle dans notre étude. Mais mérite une attention particulière quant au risque de survenue ultérieure car les facteurs de risques sont présents. Le drainage des eaux pendant les crues, les campagnes d’éducation sanitaire dans des écoles et quartiers, le respect des règles d'hygiènes permettraient de retarder la survenue des shistosomiases dans cette localité.

### Etat des connaissances actuelles sur le sujet

- Il existe un risque réel de développement de la schistosomose dans des zones irriguées, ceci a été démontré par plusieurs études;
- Non seulement Santchou est une localité autrefois aménagée de grandes surfaces de riziculture irriguée, mais aussi son relief est dominé par des plaines. Ceci explique des inondations fréquentes, facteurs qui augmentent ainsi le risque de propagation de schitosomose.

### Contribution de notre étude à la connaissance

- Notre étude montre que Santchou n'est pas actuellement une zone endémique de schistosomose;
- Toutefois, cette localité présente encore actuellement des facteurs de risque (zone de plaines, inondations, nombreux canaux, existence de mollusques hôtes intermédiaires de la schistosomiase, bains dans les eaux stagnantes par les enfants);
- Il est donc important de maintenir la sensibilisation et les mesures préventives pour éviter l'introduction de la schistosomiase dans cette localité.

## References

[1] Brooker S, Donnelly CA, Guyatt (2003). Estimating the number of helmintic infections in the republic of Cameroon from data on infection prevalence in school children. Bull World Health Organ..

[2] Ratard RC, Kouemeni LE, Ekani Bessala M, Ndamkou CN, Greer GJ, Spilsbury J (1990). Schistosomiasis in Cameroon I, distribution of schistomiasis. Am JTrop Med Hyg..

[3] Kamga GR (2003). Risque d'implantation des bilharzioses humaines dans l'aire de santé de Santchou (département de la Menoua), thèse de doctorat en médecine.

[4] Comité de Coordination du Développement Durable Santchou-cameroun-TIXIK.com..

[5] Comité national de statistique http://carpe.umd.edw/resourses/documents/reports-ccdd.pdf.

[6] Njiokou F, Onguene Onguene AR, Tchuem Tchuente LA, Kenmogne A (2004). Schistosomose urbaine au Cameroun: étude longitudinale de la transmission dans un nouveau site d'extension du foyer de schistosomose intestinale de Mélen, Yaoundé. Bull Soc Pathol Exot..

[7] Fred LN, Elúzio JL, Neci MS (2005). Comparison of the thick smear and Kato-Katz techniques for diagnosis of intestinal helminth infections. Revista da Sociedade Brasileira de Medicina Tropical..

[8] Dennis JR, Jeanette G, Michael CS (2008). Comparison of Kato–Katz Direct Smear and Sodium Nitrate Flotation for Detection of Geohelminth Infections. Comparative Parasitology..

[9] Tchuem Tchuente LA, Southgate VR, Vercruysse J (2000). La bilharziose et les géo-helminthiases dans l'arrondissement de Makénéné province du centre, Cameroun. Bull Liais Doc OCEAC..

[10] Yelnik A, Issoufa H, Appriou M, Tribouley J, Gentilini M, Ripert C (1982). Epidemiologic study of S. haematobium bilharziasis in the rice belt of Yagoua (North Cameroon) I: Prevalence of infestation and evaluation of the parasitic load. Bull Soc Pathol Exot Filiales..

[11] Folong Kamta G (2000). Etude de la schistosome génitale féminine à S haematobium et la corrélation avec la transmission du VIH dans le foyer du lac de Barombi kotto (sud-ouest Cameroun).

[12] Wibaux-Chalois M, Yelnik A, Ibrahima H, Same Ekobo A, Ripert C (1982). Etude épidémiologique de la bilharziose a S.haematobium dans le périmètre de yagoua rizicole (Nord Cameroun) II: écologie et répartition des hôtes intermédiaires. Bull Soc Pathol Exot..

[13] Yelnik A, Issoufa H, Appriou M, Tribouley J, Gentilini M, Ripert C (1982). Etude épidémiologique de la bilharziose à S.haematobium dans le périmètre de Yagoua rizicole (Nord Cameroun) I. prévalence de l'infection: évaluation de la charge parasitaire. Bull Soc Pathol Exot..

[14] Innocent T, Jean-Paul L, René M, Emmanuel N, Nicolette M, Albert SE (1993). Behavioral aspects of exposure to schistosomiasis in irrigation structures in a Sahalian area (far north Cameroon). Cahier Santé..

[15] Mayaka Ma-Nitu S (2001). Etude épidémiologie de la bilharziose à Schistosoma mansoni en milieu scolaire: Cas du groupement de Kiyanika.

[16] Stelma FF, VdWerf M, Talla I, Niang M, Gryseels B (1997). Four years’ follow-up of hepatosplenic morbidity in a recently emerged focus of Schistosoma mansoni in northern Senegal. Trans R Soc Trop Med Hyg..

